# Evaluation of cumulative prognostic score based on pretreatment plasma fibrinogen and serum albumin levels in patients with newly diagnosed high-grade gliomas

**DOI:** 10.18632/oncotarget.17849

**Published:** 2017-05-13

**Authors:** Zhen-Qiang He, Hao Duan, Chao Ke, Xiang-Heng Zhang, Cheng-Cheng Guo, Fuad Al-Nahari, Ji Zhang, Zheng-He Chen, Yin-Sheng Chen, Zhi-Gang Liu, Jian Wang, Zhong-Ping Chen, Xiao-Bing Jiang, Yong-Gao Mou

**Affiliations:** ^1^ Department of Neurosurgery/Neuro-Oncology, Sun Yat-Sen University Cancer Center, State Key Laboratory of Oncology in South China, Collaborative Innovation Center for Cancer Medicine, Guangzhou, 510060, China; ^2^ Key Laboratory of Translational Radiation Oncology, Hunan Province, Department of Radiation Oncology, Hunan Cancer Hospital, The Affiliated Cancer Hospital of Xiangya School of Medicine, Central South University, Changsha, 410013, China

**Keywords:** high-grade gliomas, fibrinogen, albumin, cumulative score, prognosis

## Abstract

This retrospective study was designed to determine the prognostic value of a cumulative score (FA score) based on pretreatment plasma fibrinogen and serum albumin levels for 326 patients newly diagnosed high-grade glioma (HGG). Receiver operating characteristic (ROC) curve analysis was performed to determine the optimal cut-off values. Univariate and multivariate analysis were performed to evaluate the independent prognostic value of the FA scores associated with overall survival (OS) and progression-free survival (PFS). The optimal cut-off values were 2.815 g/L for fibrinogen and 43.65 g/L for albumin. PFS and OS were significantly worse for patients with higher FA scores. Patients with elevated fibrinogen level and decreased albumin levels had 3.00-fold higher risk of tumor progression and had a 3.23-fold higher risk of death compared with those with normal values. Multivariate analysis demonstrated FA score was an independent predictive factor for PFS and OS. Moreover, PFS and OS were better for the patients with lower FA score, either in patients with grade III or IV gliomas. These findings indicated that the pretreatment FA score could serve as a simple and noninvasive marker to predict the prognosis of patients with HGG.

## INTRODUCTION

High-grade gliomas (HGG), defined as World Health Organization (WHO) Grade III and IV gliomas, are the most devastating malignancies in the central nerve system. HGG accounts for > 60% of all gliomas and is characterized by high morbidity and mortality because of tumor localization and locally invasive nature [1, 2]. Despite aggressive treatment modalities, including tumor resection accompanied by fractionated radiotherapy and temozolomide-based chemotherapy, the median survival for patients with glioblastoma and anaplastic gliomas are 12–14 months and 2–5 years, respectively [2, 3]. To ensure optimal adjuvant treatment and intense follow-up for patients with HGG, a simple and instructive marker is required to predict postoperative survival.

The histopathological characteristics, performance status, tumor grade, prognostic indicators based on systemic inflammation levels and nutritional conditions of patients with HGG are published [4, 5]. Fibrinogen, a 340-kD liver glycoprotein, has been reported as a key regulator in both inflammation and cancer progression [6]. Moreover, fibrinogen plays an important role in tumor cell proliferation, migration and angiogenesis [6]. Further, high pretreatment plasma fibrinogen levels are significantly associated with shorter survival in rectal cancer, non-small cell lung cancer, renal cell carcinoma and breast cancers [7–10]. Serum albumin levels are an important indicator of host's systemic inflammatory response and nutritional status as a prognostic indicator in several cancer [11–13]. Moreover, low serum albumin levels are associated with significantly short survival in glioblastoma patients [14].

The fibrinogen and albumin score (FA score), a novel cumulative prognostic score based on pretreatment fibrinogen and albumin levels, was firstly proposed by Matsuda et al. as a new prognostic scoring system in esophageal cancer patients treated with transthoracic esophagectomy [15]. The FA score is of outstanding prognostic value in esophageal cancer patients compared with Glasgow Prognostic Score (GPS) [15]. However, whether FA score predicts the survive of patients with HGG is unknown. We therefore performed a retrospective analysis to evaluate the prognostic performance of the FA score in a relatively large cohort of patients with HGG.

## RESULTS

### Patient characteristics

The characteristics of 326 HGG patients are shown in Table 1. The median follow-up was 20.0 months (interquartile range [IQR]: 13.2–36.2 months), mean age was 44.0 years (range: 5–78 years), and 60.4% (197/326) of patients were males. Gross total resection (GTR) and subtotal resection (STR) was achieved for 63.5% and 27.3% of patients, respectively, and 8.6% underwent partial resection and 0.6% patients received biopsy only. Aggressive adjuvant treatment of 179 patients (54.9%) included fractionated radiotherapy and first-line chemotherapy. The treatment modalities administered to patients were summarized in Table 2.

**Table 1:** The clinicopathological features stratified by pretreatment FA score (*n* = 326)

Variables	All patients	FA score 0 (*n* = 79)	FA score 1 (*n* = 171)	FA score 2 (*n* = 76)	*P* value
Age [years; *n* (%)]					< 0.001
< 60	269 (82.5)	75 (27.9)	140 (52.0)	54 (20.1)	
≥ 60	57 (17.5)	4 (7.0)	31 (54.4)	22 (38.6)	
Gender [*n* (%)]					0.286
Male	198 (60.7)	37 (28.9)	63 (49.2)	28 (21.9)	
Female	128 (39.3)	42 (21.2)	108 (54.5)	48 (24.2)	
KPS [*n* (%)]					0.727
≥ 70	303 (92.9)	75 (24.8)	158 (52.1)	70 (23.1)	
*<* 70	23 (7.1)	4 (17.4)	13 (56.5)	6 (26.1)	
Tumor grade [*n* (%)]					0.007
WHO III	157 (48.2)	48 (30.6)	82 (52.2)	27 (17.2)	
WHO IV	169 (51.8)	31 (18.3)	89 (52.7)	49 (29.0)	
Tumor size [cm; *n* (%)]					0.220
≤ 5	156 (47.9)	42 (26.9)	74 (47.4)	40 (25.6)	
> 5	170 (52.1)	37 (21.8)	97 (57.1)	36 (21.2)	
Tumor location [*n* (%)]					0.079
Cerebral cortex	291 (89.3)	66 (22.7)	153 (52.6)	72 (24.7)	
Non cerebral cortex	35 (10.7)	13 (37.1)	18 (51.4)	4 (11.4)	
Extent of resection [*n* (%)]					0.829
Gross total resection	207 (63.5)	52 (25.1)	108 (52.2)	47 (22.7)	
Subtotal resection	89 (27.3)	18 (20.2)	49 (55.1)	22 (24.7)	
Partial resection and biopsy	30 (9.2)	9 (30.0)	14 (46.7)	7 (23.3)	
Aggressive treatment [*n* (%)]					0.122
Yes	183 (56.1)	48 (26.2)	100 (54.6)	35 (19.1)	
No	143 (43.9)	31 (21.7)	71 (49.7)	41 (28.7)	
Pretreatment plasma fibrinogen level [g/L; mean ± SD]	2.97 ± 1.06	2.34 ± 0.30	2.90 ± 1.13	3.78 ± 0.87	< 0.001
Pretreatment serum albumin level [g/L; mean ± SD]	43.37 ± 3.80	46.74 ± 2.85	43.09 ± 3.54	40.52 ± 2.28	< 0.001

FA score, fibrinogen and albumin score; n, number; KPS, Karnofsky performance status; WHO, World Health Organization;

**Table 2:** Treatment modality for the patients (*n* = 326)

Treatment modality	*N* (%)
Surgical resection only	104 (31.9)
Surgery→Radiotherapy	32 (9.8)
Surgery→Radiotherapy→Chemotherapy (A)	129 (39.6)
Surgery→Radiotherapy→Chemotherapy (B)	50 (15.3)
Surgery→Chemotherapy (A)	6 (1.8)
Surgery→Chemotherapy (B)	5 (1.5)

A, Temozolomide-based; B, nitrosourea-based or platinum-based.

### Cut-off determination of fibrinogen and albumin

The mean pretreatment plasma fibrinogen and serum albumin levels were 2.97 ± 1.06 g/L (range: 1.39–13.00 g/L) and 43.37 ± 3.80 g/L (range: 32.90–64.39 g/L). The areas under the receiver operating characteristic (ROC) curve were 0.611 for fibrinogen and 0.605 for albumin. Using ROC analysis, the optimal cut-off values for fibrinogen was 2.815 g/L and 43.65 g/L for albumin, respectively. Among the 326 patients, 151 (46.3%) patients had elevated pretreatment fibrinogen levels ≥2.815 g/L, and 172 (52.8%) patients had decreased pretreatment albumin levels ≤ 43.65 g/L. Therefore, 79 (24.2%), 171 (52.5%), and 76 (23.3%) patients had FA scores = 0,1 and 2, respectively (Table 1).

### Association between clinicopathological features and FA score

The clinicopathological features of all patients stratified according to FA scores are shown in Table 1. Higher pretreatment FA score were observed for patient of advanced age (*p* < 0.001) and a higher glioma grade (*p* < 0.007). However, there was no significant association between FA score and sex, Karnofsky performance status (KPS), tumor size, tumor location, extent of resection, or treatment modality.

### Survival analysis

**Figure 1:**
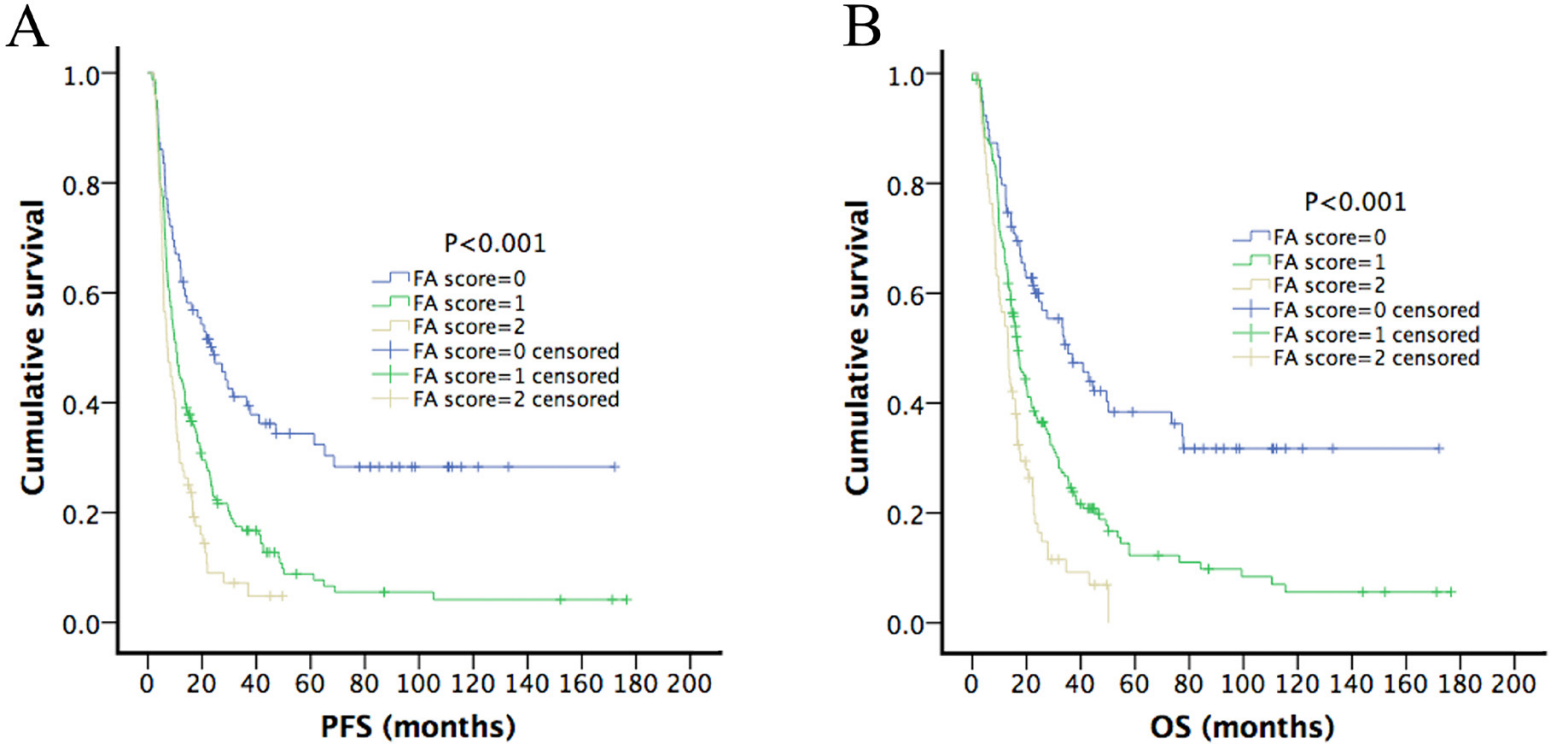
Kaplan-Meier survival curves of HGG patients According to the optimal cut-off value of pretreatment plasma fibrinogen and serum albumin levels, we build up a cumulative score (FA score) and HGG patients were divided into three groups. Patients with elevated fibrinogen level (≥ 2.815 g/L) and decreased albumin level (≤ 43.65 g/L) were allocated to a score of 2, those with only one of these abnormalities were allocated to a score of 1 and those with neither of these abnormalities were allocated a score of 0. Pretreatment FA score is significantly predictive of DFS and OS, with higher FA scores among patients with shorter PFS (**A**, *P* < 0.001) and OS (**B**, *P* < 0.001).

**Figure 2:**
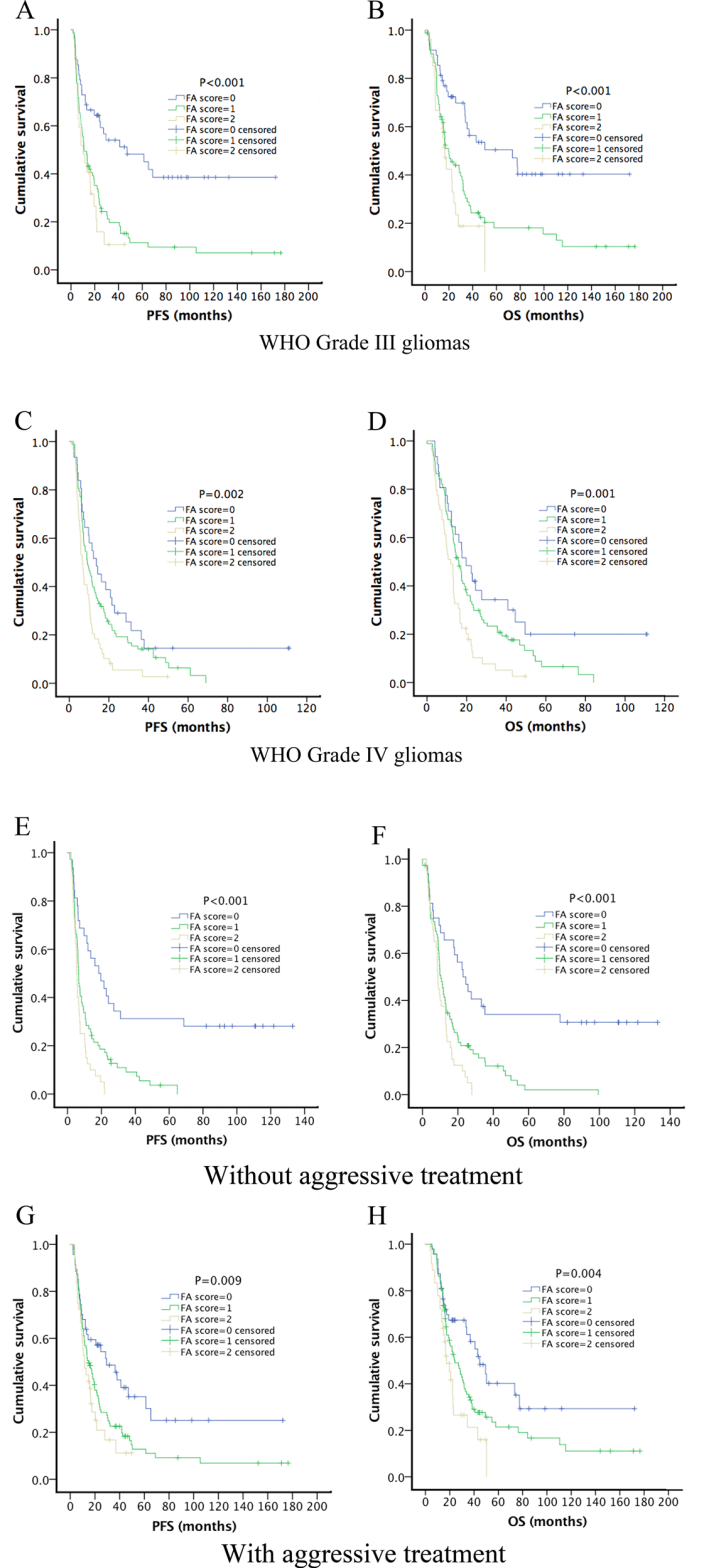
Kaplan-Meier survival curves of different HGG subgroups Kaplan-Meier method and log-rank test were used to investigate the prognostic value of pretreatment FA score in each subgroup. High FA score was significantly associated with better PFS and OS in subgroups of WHO grade III gliomas (**A**, PFS: *P* < 0.001; **B**, OS: *P* < 0.001) and WHO grade IV gliomas (**C**, PFS: *P* = 0.002; **D**, OS: *P* = 0.001). Subgroup analysis based on treatment modalities showed that high pretreatment FA score still significantly predicted worse PFS and OS in patients received aggressive treatment (**E**, PFS, *P* = 0.009; **F**, OS, *P* = 0.004) or patients without aggressive treatment (**G**, PFS, *P* < 0.001; **H**, OS, *P* < 0.001).

At the date of final follow-up, 254 (77.9%) patients had died. The median OS of all the patients was 17.23 months (95% CI = 15.14–19.33 months), and 25.53 months (95% CI = 17.03–34.04 months) and 14.30 months (95% CI = 12.22–16.38 months) for those with WHO Grades III and IV gliomas, respectively. Kaplan-Meier analysis revealed that that the pretreatment FA score was negatively associated with PFS (*p* < 0.001) and OS (*p* < 0.001) (Figure 1). When we conducted subgroup analysis of prognosis after stratifying patients according to tumor grade, we found that the pretreatment FA score retained its predictive value for PFS and OS for patients with grades III (PFS, *p* < 0.001; OS, *p* < 0.001) and IV (PFS, *p* = 0.002; OS, *p* = 0.001) disease, respectively (Figure 2A–2D). We next performed Kaplan–Meier analysis of patients stratified according to treatment modalities. As shown in Figure 2E–2H, a high pretreatment FA score significantly predicted worse PFS and OS of patients who did (PFS, *p* = 0.009; OS, *p* = 0.004) or did not (PFS, *p* < 0.001; OS, *p* < 0.001) receive aggressive treatment.

### Univariate and multivariate Cox regression analysis

Univariate analysis revealed that pretreatment FA score, as well as traditionally predictors including age, KPS, tumor grade, extent of resection and aggressive treatment were significantly associated with both OS and PFS (Table 3), and patients with FA score = 2 had 3.00-fold higher risk of tumor progression and a 3.23-fold higher risk of death compared with those with FA score = 0. Furthermore, multivariate Cox regression analysis revealed that higher pretreatment FA score still predicted worse PFS (*p* < 0.001) and OS (*p* < 0.001) independent of age, tumor grade, extent of resection or aggressive treatment (Table 4).

**Table 3:** The univariate analysis of the prognostic factors for PFS and OS (*n* = 326)

	Progression-free survival			Overall survival		
**HR**	**95% CI**	***P* value**	**HR**	**95% CI**	***P* value**
Age, years			*<* 0.001			< 0.001
< 60	1	Referent		1	Referent	
≥ 60	2.184	1.614–2.954		2.095	1.537–2.854	
Gender			0.039			0.140
Male	1	Referent		1	Referent	
Female	0.770	0.601–0.987		0.826	0.640–1.065	
KPS			*<* 0.001			0.002
≥ 70	1	Referent		1	Referent	
*<* 70	2.014	1.308–3.103		1.966	1.277–3.026	
Tumor grade			*<* 0.001			< 0.001
WHO grade III	1	Referent		1	Referent	
WHO grade IV	1.676	1.313–2.139		1.772	1.375–2.283	
Tumor size			0.277			0.201
≤ 5 cm	1	Referent		1	Referent	
**>** 5 cm	0.876	0.690–1.112		0.851	0.666–1.089	
Tumor location			0.065			0.072
Cerebral cortex	1	Referent		1	Referent	
Non cerebral cortex	1.409	0.987–2.030		1.413	0.969–2.058	
Extent of resection			*<* 0.001			< 0.001
Gross total resection	1	Referent		1	Referent	
Subtotal resection	1.656	1.268–2.163	0.001	1.587	1.206–2.090	0.001
Partial resection and biopsy	3.734	2.486–5.607	*<* 0.001	3.536	2.330–5.365	< 0.001
Aggressive treatment			*<* 0.001			< 0.001
Yes	1	Referent		1	Referent	
No	1.889	1.486–2.403		2.224	1.736–2.850	
FA score			*<* 0.001			< 0.001
0	1	Referent		1	Referent	
1	2.017	1.464–2.777	*<* 0.001	1.988	1.424–2.776	< 0.001
2	2.996	2.064–4.350	*<* 0.001	3.233	2.198–4.754	< 0.001

HR, hazard ratio; CI, confidence interval; KPS, Karnofsky performance status; WHO, World Health Organization; FA score, fibrinogen and albumin score

**Table 4:** The multivariate analysis of the prognostic factors for PFS and OS (*n* = 326)

	Progression-free survival			Overall survival		
**HR**	**95% CI**	***P*** **value**	**HR**	**95% CI**	***P*** **value**
Age, years			0.003			0.015
< 60	1	Referent		1	Referent	
≥ 60	1.614	1.175-2.218		1.496	1.080-2.073	
KPS			0.102			0.152
≥ 70	1	Referent		1	Referent	
< 70	1.460	0.928-2.301		1.393	0.885-2.194	
Tumor grade			< 0.001		< 0.001	< 0.001
WHO grade III	1	Referent		1	Referent	
WHO grade IV	1.593	1.234-2.055		1.715	1.318-2.232	
Extent of resection			< 0.001			< 0.001
Gross total resection	1	Referent		1	Referent	
Subtotal resection	1.613	1.222-2.129	0.001	1.562	1.171-2.084	0.002
Partial resection and biopsy	5.324	3.467-8.175	< 0.001	5.165	3.331-8.008	< 0.001
Aggressive treatment			< 0.001			< 0.001
Yes	1	Referent		1	Referent	
No	2.059	1.608-2.637		2.457	1.905-3.169	
FA score			< 0.001			< 0.001
0	1	Referent		1	Referent	
1	2.041	1.464-2.846	< 0.001	2.046	1.447-2.894	< 0.001
2	2.792	1.890-4.123	< 0.001	3.032	2.026-4.536	< 0.001

HR, hazard ratio; CI, confidence interval; KPS, Karnofsky performance status; WHO, World Health Organization; FA score, fibrinogen and albumin score

## DISCUSSION

In the present study, we retrospectively analyzed 326 consecutive patients with high-grade gliomas who were treated with surgery and adjuvant therapies. Using the cut-off values of pretreatment fibrinogen and albumin determined from ROC curves, we examined the prognostic value of a cumulative prognostic score (pretreatment FA score). The results are as follows: (1) A higher pretreatment FA score was significantly associated with advanced age and higher tumor grade. (2) The pretreatment FA score was an independent prognostic factor of PFS and OS of patients with high-grade gliomas. (3) The prognostic value of the FA score was significant in subgroups that included patients diagnosed with WHO Grade III or IV as well as patients who did or did not undergo aggressive treatment.

This novel cumulative prognostic score proposed by Matsuda et al is based on pretreatment plasma fibrinogen and serum albumin levels [15] which serve as prognostic markers of solid cancers [6, 13]. The adverse effect of elevated pretreatment plasma fibrinogen on survival is experienced in patients with colon cancer[11], lung cancer [8], breast cancer [10], renal cell carcinoma[16] and ovarian cancer [17]. However, the prognostic value of pretreatment fibrinogen levels in patients with high-grade glioma was previously unknown. In contrast, pretreatment serum albumin levels are prognostic for patients with glioblastoma. Schwartzbaum et al and Borg et al reported that a low preoperative serum albumin level is a significant predictor of poor overall survival in patients with glioblastoma multiforme (GBM) [14, 18].

Here we show that the FA score showed significant prognostic value in HGG patients. Patients with a FA score = 1 had 2.406-fold higher risk of death than those with a FA score of 0. And patients with a FA score of 2 had a 3.032-fold higher risk of death than those with a FA score = 0 in the multivariate analysis. Therefore, HGG patients with a higher FA score before surgery may require aggressive treatment and intense follow-up.

Although the prognostic value of the pretreatment albumin level and fibrinogen level have been established in patients with cancers [8, 10–11, 16–17], the mechanisms responsible for these associations are unknown. Fibrinogen detected using plasma is an important marker of systemic inflammatory. Fibrinogen can enhance tumor progression by inducing tumor cell proliferation, migration, and angiogenesis [19–21]. Fibrinogen is a common component of fibrinogen/fibrin matrix surrounding tumor cells, serving as a scaffold for binding members of growth factor families, such as transforming growth factor-β (TGF-β), vascular endothelial growth factor (VEGF), fibroblast growth factor (FGF) and platelet-derived growth factor (PDGF) [22]. The binding of growth factors promotes tumor proliferation and stimulates angiogenesis [23].

Fibrinogen plays an important role in metastasis as well as facilitating stable adhesion, survival of metastatic emboli, or both, after tumor intravasation [24]. Fibrinogen deficiency can significantly diminish spontaneous hematogenous and lymphatic metastasis in the mice bearing levis lung carcinoma [24]. Adams et al reported that fibrinogen can support tumor growth as well as local invasion and metastasis, which contribute to the pathogenesis of colonic adenocarcinoma [25]. Moreover, fibrinogen may modulate the inflammatory response by inducing the production by peripheral blood mononuclear cells of the pro-inflammatory cytokines interleukin-1 (IL-1), interleukin-6 (IL-6), and tumor necrosis factor-α (TNF-α) [26]. Moreover, inflammatory responses induced by fibrinogen in the tumor microenvironment are implicated in cancer progression [27].

In contrast, the level of circulating IL-6 was significantly higher in GBM patients compared with those of normal controls [28]. IL-6 is implicated in the regulation of VEGF secretion from glioblastoma cells, and VEGF can induce vascular permeability, which contributes to decreasing serum albumin levels [29]. Pro-inflammatory cytokines such as IL-1, IL-6 and TNF-α can down-regulate the hepatic synthesis of albumin [30]. The serum albumin level is inversely related to the host's systemic inflammatory response and nutritional status [31]. Therefore, a reduced albumin level may diminish the response of cancer patients to treatment and indicates poor survival. For these reasons, the FA score may serve as a prognostic indicator for patients with HGG.

Our study had certain limitations. First, this is a retrospective analysis with a single-center design, which may introduce selection bias. Further, the prognostic value of the FA score was not verified using a validation cohort. Second, data were unavailable for *IDH1/2* mutations, codeletion of chromosomes 1p and 19q, *TERT* promoter mutations and *ATRX* mutation, which are prognostic factors for HGG [32]. Finally, because of its respective design, the PFS data might not be accurate. However, quantification of PFS is very difficult even in prospective trials because of pseudo-progression. Despite its limitations, we conclude that our study demonstrates the FA score serves as a significant prognostic indicator for managing patients with HGG. However, our findings must be validated by prospective trials.

In conclusion, we demonstrate for the first time that higher FA scores can significantly predict worse PFS and OS of patients with HGG. Fibrinogen and albumin levels are routinely measured in clinical practice. Thus, the FA score is an easily determined, noninvasive, economical indicator, which can facilitate predicting prognosis and guiding the administration of individualized treatment to patient with high-grade gliomas.

## MATERIALS AND METHODS

### Ethical statement

All patients have provided written informed consent for their information to be stored and used in the hospital database. Study approved was obtained from the Medical Ethics Committee of Sun Yat-sen University Cancer Center and the study was conducted in accordance with the ethical standard of the World Medical Association Declaration of Helsinki [33].

### Study population and data collection

The inclusion criteria were as follows: (1) confirmation of WHO Grade III or IV glioma, (2) absence of other malignancies, (3) absence of other treatments before admission, and (4) integrity of clinical information and followed-up data. The exclusion criteria were as follows: (1) acute infection or chronic active inflammatory diseases, (2) glucocorticoid treatment before admission and (3) perioperative surgery-related mortality. Accordingly, we enrolled and retrospectively reviewed the records of 326 patients with histologically diagnosed high-grade gliomas who underwent tumor resection at Sun Yat-sen University Cancer Center from January 1 2001 to July 31 2014.

Baseline characteristics including demographics, KPS, pathological diagnosis, tumor grade, tumor size, tumor location, extent of resection and therapeutic information were collected using an electronic medical record system. All pathological specimens were reviewed and reclassified by central review according to WHO classification (revised in 2007) of central nervous system tumors [34]. High-grade gliomas were defined as WHO Grade III and IV gliomas. The tumor size was defined as the maximum diameter measured using pretreatment enhanced T1-weighted magnetic resonance imaging (MRI). For patients with multiple lesions, tumor size was calculated by adding the long diameter of all enhanced focus. Tumor location was categorized as cerebral cortex and non-cerebral cortex areas.

### Treatment and follow up

All patients underwent tumor resection at Sun Yat-sen University Cancer Center and the extent of resection was classified as GTR, STR, partial resection and biopsy according to postoperative MRI and surgeon's notes. Aggressive adjuvant treatment was defined as fractionated radiotherapy plus adjuvant first-line chemotherapy or chemoradiotherapy plus adjuvant first-line chemotherapy post-operatively. First-line chemotherapy was categorized as temozolomide-based and nitrosourea/platinum-based strategies. A patient's refusal, financial problems, or poor condition excluded administration of aggressive treatment.

For all the patients, follow-up started from the date of surgery. Patients were generally followed quarterly for the first year, semiannually for the following 2 years and annually thereafter. During follow-up, patents were encouraged to undergo repeat contrast-enhanced MRI. Recording of medical history and physical examination were routinely performed. The last follow-up was until October 31, 2015 included verification of the clinical attendance records and direct telecommunications with the patients or their families. OS was measured from the date of surgery to the date of death from any cause, or the date of last follow-up visit. PFS was calculated from surgery to the first detection of progression, relapse, death from any cause, or the date of the last follow-up visit. Progression or relapse was identified according to the latest radiographic evidence.

### FA score

Pretreatment plasma fibrinogen and serum albumin levels were determined using fasting serum sample after admission. The fibrinogen and albumin cut-off value were determined from receiver operating characteristic curves. Patients with elevated fibrinogen and decreased albumin levels were allocated to a score = 2, those with one abnormal level were allocated to a score = 1 and those with neither abnormal levels were allocated a score = 0.

### Statistical analysis

Differences of baseline and clinicopathological parameters between groups were evaluated by chi-square test or Fisher's exact test according to the distribution of data. The survival curves were calculated using the Kaplan-Meier method. Differences between the curves were evaluated using the log-rank test. The significant parameters identified by univariate analysis were evaluated using multivariate analysis employing the Cox proportional hazards model. All reported *p* values were two-sided. *p* < 0.05 was considered significant, and 95% CIs were calculated. All analyses were performed using the SPSS Statistics version 22.0 (IBM Corporation, Armonk, NY, United States).
